# Donor Site Location Is Critical for Proliferation, Stem Cell Capacity, and Osteogenic Differentiation of Adipose Mesenchymal Stem/Stromal Cells: Implications for Bone Tissue Engineering

**DOI:** 10.3390/ijms19071868

**Published:** 2018-06-26

**Authors:** Marie K. Reumann, Caren Linnemann, Romina H. Aspera-Werz, Sigrid Arnold, Manuel Held, Claudine Seeliger, Andreas K. Nussler, Sabrina Ehnert

**Affiliations:** 1 Department of Trauma and Reconstructive Surgery, Siegfried Weller Research Institute, Eberhard Karls University Tuebingen, BG Trauma Center Tuebingen, 72076 Tuebingen, Germany; mreumann@bgu-tuebingen.de (M.K.R.); caren.linnemann@student.uni-tuebingen.de (C.L.); rominaaspera@hotmail.com (R.H.A.-W.); sigrid-arnold@gmx.de (S.A.); mheld@bgu-tuebingen.de (M.H.); sabrina.ehnert@gmail.com (S.E.); 2 Department of Hand, Plastic, Reconstructive and Aesthetic Surgery, Eberhard Karls University Tuebingen, BG Trauma Center Tuebingen, 72076 Tuebingen, Germany; 3 Else Kröner-Fresenius Center for Nutritional Medicine, Technical University of Munich, 85354 Freising, Germany; claudine.seeliger@tum.de; 4 Experimental Trauma Surgery, Klinikum Rechts der Isar, Technical University of Munich, 81675 Munich, Germany

**Keywords:** primary human adipose-derived mesenchymal stem/stromal cells (Ad-MSCs), proliferation, AP activity, matrix mineralization

## Abstract

Human adipose mesenchymal stem/stromal cells (Ad-MSCs) have been proposed as a suitable option for bone tissue engineering. However, donor age, weight, and gender might affect the outcome. There is still a lack of knowledge of the effects the donor tissue site might have on Ad-MSCs function. Thus, this study investigated proliferation, stem cell, and osteogenic differentiation capacity of human Ad-MSCs obtained from subcutaneous fat tissue acquired from different locations (abdomen, hip, thigh, knee, and limb). Ad-MSCs from limb and knee showed strong proliferation despite the presence of osteogenic stimuli, resulting in limited osteogenic characteristics. The less proliferative Ad-MSCs from hip and thigh showed the highest alkaline phosphatase (AP) activity and matrix mineralization. Ad-MSCs from the abdomen showed good proliferation and osteogenic characteristics. Interestingly, the observed differences were not dependent on donor age, weight, or gender, but correlated with the expression of *Sox2*, *Lin28A*, *Oct4α*, and *Nanog*. Especially, low basal *Sox2* levels seemed to be pivotal for osteogenic differentiation. Our data clearly show that the donor tissue site affects the proliferation and osteogenic differentiation of Ad-MSCs significantly. Thus, for bone tissue engineering, the donor site of the adipose tissue from which the Ad-MSCs are derived should be adapted depending on the requirements, e.g., cell number and differentiation state.

## 1. Introduction

The regeneration of large bone defects after a trauma or tumor surgery is still a major issue in orthopedic surgery [1]. Treatment strategies require bone fixation in order to stabilize critical-size bone defects as well as to fill the structural gaps to allow bone bridging and, finally, healing [2]. Highly specialized surgical techniques have been deployed in order to reconstitute large bone defects, ranging from microvascular anastomosed autogenous bone grafting (fibula transfer flap) to Masquelet technique with Ilizarov external fixation and bone segmental transport. However, these methods require large amounts of bony tissue to fill the defects and provide mesenchymal stem/stromal cells (MSCs), which are essential to induce bone healing [3,4]. The most frequently used autologous bone grafting requires additional surgical intervention. However, this carries the risk of infection as well as morbidity at the donor site [5]. On the other hand, allogeneic bone tissue, which might serve as an alternative, can cause immunological reactions [6].

A possible alternative to bone tissue transplantation is alloplastic replacement with synthetic bone-filling materials. These materials, mainly used to fill the defect area, are then encapsulated in the connective tissue. Lacking osteoinductive capacity, these filling materials carry the risk of failing. Thus, tissue engineering with MSCs offers the advantage of providing large amounts of biologically active bone tissue to stimulate ossification and finally healing of critical-size bone defects [4]. Up to now, there are more than 350 clinical trials reported, which investigate the cell therapeutic potential of MSCs [7].

MSC are multipotent cells with high regenerative capacity and can be differentiated into various cell types, e.g., osteoblasts, chondrocytes, and adipocytes [8]. In addition to their potential of direct cell differentiation into the target tissue, MSCs trigger alternative mechanisms, such as secretion of growth factors, cytokines, or hormones. Also, MSCs interact with other cells by forming nanochannels or releasing extracellular vesicles and exosomes that contain proteins, mRNAs, or miRNAs. Thus, MSCs stimulate cell proliferation, reduce apoptosis, and, in some cases, even regulate the immune response [9].

Usually, for bone regeneration, MSCs are derived from bone marrow aspirates (B-MSCs). However, only limited amounts can be harvested to reduce the side effects, i.e., anemia [10]. A possible alternative is deriving MSCs from the adipose tissue (Ad-MSCs), which contains large amounts of stem cells [11] that can be obtained at lower risk (minimal invasive techniques) for the patient [12,13]. However, it is often critically discussed that Ad-MSCs possess only a limited osteogenic differentiation potential. Though transplanted undifferentiated Ad-MSCs and B-MSCs can be stimulated to bone tissue formation [14,15], the newly formed bone tissue may show weaknesses in its mechanical properties: the mechanical stability (maximum load and elasticity) of scaffolds with Ad-MSCs was shown to be reduced compared to that of scaffolds with B-MSCs [16]. A reason for this, however, could be the support materials themselves, which are mostly flexible in order to transmit mechanical stimuli to the cells [17]. Former studies could show that donor age as well as cell culture duration adversely affect the osteogenic differentiation potential of Ad-MSCs via epigenetic changes [18,19,20,21]. The altered epigenetics affects the differentiation capacity by regulating the expression of transcription factors (*Sox2*, *Lin28A*, *Oct4α*, and *Nanog*) involved in the self-renewal of embryonic stem cells and the generation of induced pluripotent stem cells [21]. An increasing donor age is often associated with a decreased expression of *Nanog, Oct4α*, and *Lin28A* [21].

So far, little is known about the influence of the donor site on the osteogenic differentiation potential of Ad-MSCs. Therefore, the aim of this study was to compare the proliferation (increase in total protein content) and osteogenic differentiation potential (AP activity, matrix mineralization, expression of molecular markers (osteogenic transcription factors)) of primary human Ad-MSCs derived from adipose tissue obtained from different donor sites (abdomen, hip, thigh, knee, and limb). Furthermore, to assess the stem cell capacity of each donor site, we measured the expression levels of *Sox2*, *Lin28A*, *Oct4α*, and *Nanog*.

## 2. Results

### 2.1. The Proliferation of Ad-MSCs Varies Depending on the Donor Site

**Figure 1:**
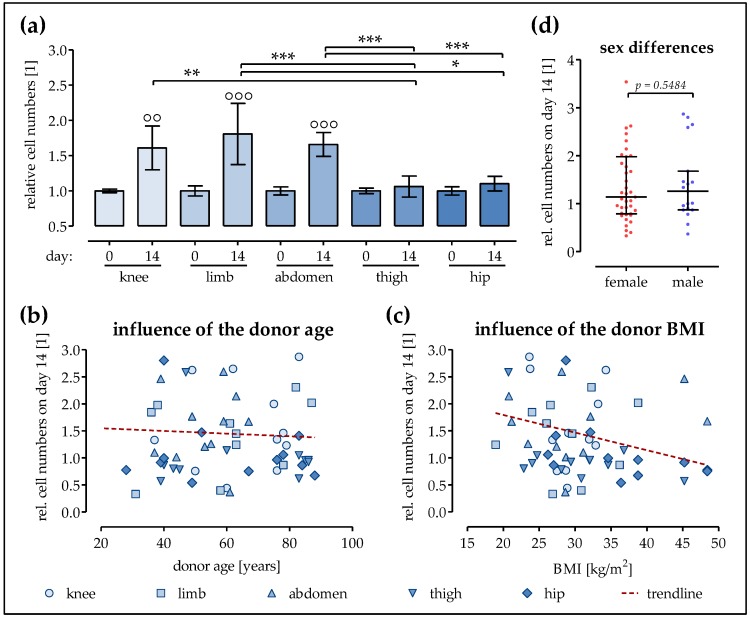
The proliferation of mesenchymal stem/stromal cells from adipose tissue (Ad-MSCs) varies depending on the donor site. Ad-MSCs derived from adipose tissue of the knee (*N* = 12), limb (*N* = 11), abdomen (*N* = 11), thigh (*N* = 12), and hip (*N* = 12) were osteogenically differentiated for 14 days. (**a**) To determine cell proliferation, the total protein content was determined by Sulforhodamine B (SRB) staining on days 0 and 14 of differentiation; (**b**) Relative cell numbers on day 14 of differentiation as a function of the donor age; (**c**) Relative cell numbers on day 14 of differentiation as a function of the donor body mass index (BMI); (**d**) Comparison of the relative cell numbers of differentiated (day 14) Ad-MSCs from male and female donors; °° *p* < 0.01 and °°° *p* < 0.001 when comparing day 0 with day 14 within each group; * *p* < 0.05, ** *p* < 0.01, and *** *p* < 0.001 as indicated.

No significant differences were found in the number of Ad-MSCs obtained after collagenase digestion of fat tissues derived from different donor sites (abdomen, hip, thigh, knee, and limb). Cell expansion till passage three lasted for 3 to 4 weeks in all cells and was mainly dependent on the initial amount of cells. To compare the proliferation potential of Ad-MSCs derived from different donor sites, the cells were plated at a density of 10,000 cells/cm^2^ and cultured for 14 days in osteogenic differentiation medium. Immediately after plating, no significant difference in the amounts of cells could be detected, as measured by Sulforhodamine B (SRB) staining. Within the following 14 days, the amount of Ad-MSCs originally derived from the hip and thigh hardly increased (10% and 6%, respectively). However, significant increases in the cell amount of about 61% (*p* < 0.01) and 65% (*p* < 0.001) were detected in the Ad-MSCs from the knee or abdomen, respectively. In addition, the Ad-MSCs from the limb showed the most extensive growth (Figure 1a) by increasing up to 81%. As listed in Table 1, the average donor age as well as the gender distribution within the groups varied. In order to rule out variations based on donor age, the next step was to investigate the influence of donor age on proliferation. Comparing all donors—irrespective of fat tissue location—the proliferation potential of Ad-MSCs did not decrease significantly with the increase of the donor age (slope = −0.0025 ± 0.0069, Figure 1b). Similarly, with increases in donor body mass index (BMI) (irrespective of fat tissue location), only a slight decrease (slope = −0.0326 ± 0.0169, Figure 1c) in cell proliferation was observed. In addition, no significant difference in the proliferation potential of Ad-MSCs was observed with respect to the donor gender (Figure 1d).

### 2.2. Strongest Increase in AP Activity in Ad-MSCs of the Hip and Thigh

**Figure 2:**
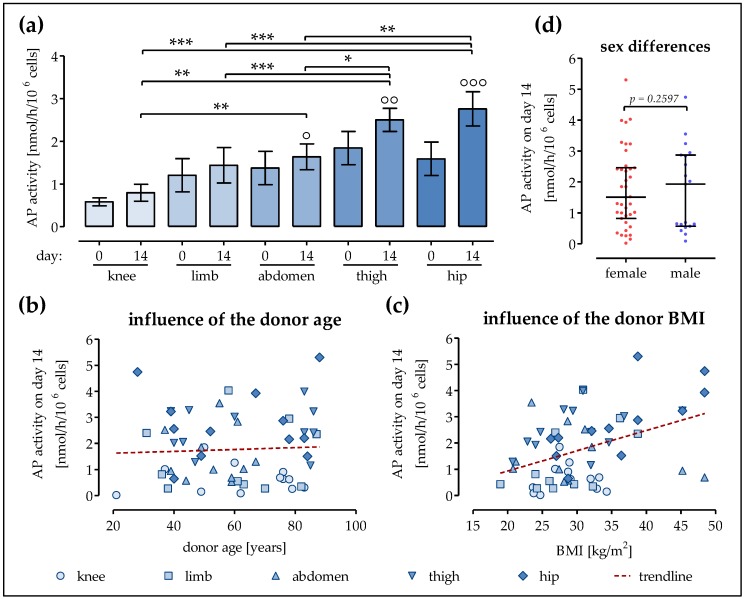
The alkaline phosphatase (AP) activity of Ad-MSCs varies depending on the donor site. Ad-MSCs derived from adipose tissue of the knee (*N* = 12), limb (*N* = 11), abdomen (*N* = 11), thigh (*N* = 12), and hip (*N* = 12) were osteogenically differentiated for 14 days. (**a**) AP activity was determined on days 0 and 14 of differentiation; (**b**) AP activity on day 14 of differentiation as a function of the donor age; (**c**) AP activity on day 14 of differentiation as a function of the donor BMI; (**d**) Comparison of the AP activity of differentiated (day 14) Ad-MSCs from male and female donors; ° *p* < 0.05, °° *p* < 0.01, and °°° *p* < 0.001 when comparing day 0 with day 14 within each group; * *p* < 0.05, ** *p* < 0.01, and *** *p* < 0.001 as indicated.

In order to determine the osteogenic differentiation potential of Ad-MSCs derived from different donor sites, plated (10,000 cells/cm^2^) cells were osteogenically differentiated for 14 days. Immediately after plating, the highest AP activity (2.46 ± 0.27 nmol/h/10^6^ cells) was measured in Ad-MSCs derived from the thigh, followed by Ad-MSCs of the abdomen (1.99 ± 0.31 nmol/h/10^6^ cells), extremities (1.84 ± 0.19 nmol/h/10^6^ cells), and hip (1.59 ± 0.19 nmol/h/10^6^ cells). The lowest basal AP activity (0.58 ± 0.05 nmol/h/10^6^ cells) was measured in the Ad-MSCs of the knee. After 14 days of differentiation, a significant increase in AP activity (1.2- to 1.7-fold) was found only in Ad-MSCs of the abdomen, hip, and thigh (Figure 2a). Similar to the SRB staining results, the AP activity did not decrease significantly with increases in donor age (slope = 0.0034 ± 0.0096, Figure 2b), when comparing all donors irrespective of the fat tissue location. Similarly, we observed only a slight increase in AP activity (slope = 0.0769 ± 0.0218, insignificant, Figure 2c) with increases in donor BMI (irrespective of fat tissue location). The sex of the donors also had no significant influence on AP activity (Figure 2d).

### 2.3. Strongest Matrix Mineralization in Ad-MSCs of the Hip and the Thigh

**Figure 3:**
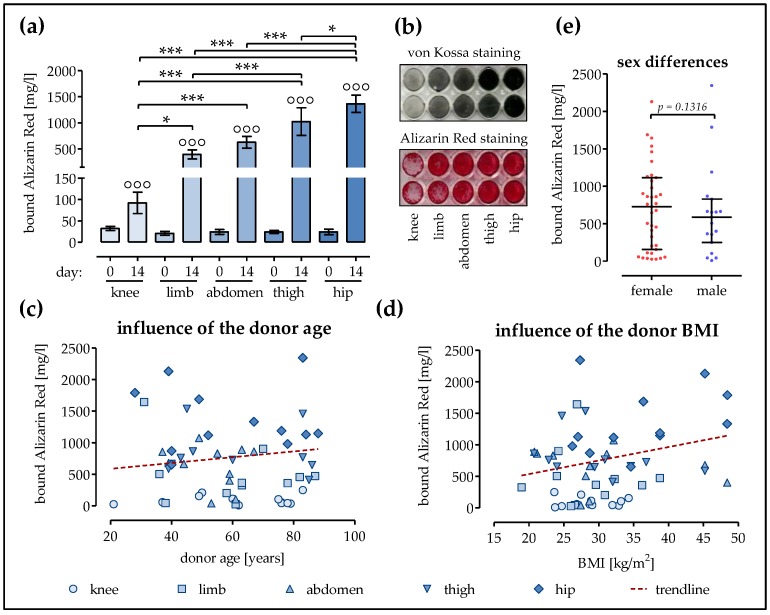
The formation of mineralized matrix by Ad-MSCs varies depending on the donor site. Ad-MSCs derived from adipose tissue of the knee (*N* = 12), limb (*N* = 11), abdomen (*N* = 11), thigh (*N* = 12), and hip (*N* = 12) were osteogenically differentiated for 14 days. (**a**) Matrix mineralization was quantified by Alizarin Red staining on days 0 and 14 of differentiation; (**b**) Representative scan of von Kossa- and Alizarin Red-stained Ad-MSCs from the different donor sites; (**c**) Mineralized matrix formed by differentiated (day 14) Ad-MSCs as a function of the donor age; (**d**) Mineralized matrix formed by differentiated (day 14) Ad-MSCs as a function of the donor BMI; (**e**) Comparison of the matrix mineralization of differentiated (day 14) Ad-MSCs from male and female donors; °°° *p* < 0.001 when comparing day 0 with day 14 within each group; * *p* < 0.05 and *** *p* < 0.001 as indicated.

The formation of mineralized matrix was determined by von Kossa and Alizarin Red staining for late markers for osteogenesis. As expected, there were no significant differences in matrix mineralization at day 0 (average 22.44 ± 2.12 mg/L or Alizarin Red stain). After 14 days of osteogenic differentiation, tough, a significant increase in matrix mineralization was observed in all Ad-MSCs. The smallest increases were seen in Ad-MSCs of the knee (2.9-fold) and limb (18.9-fold). Significantly increased matrix mineralization was observed in Ad-MSCs from the abdomen (25.8-fold increase) and the thigh (42.2-fold increase). The strongest mineralized matrix was observed in Ad-MSCs of the hip (56.5-fold increase, Figure 3a,b). Similar to AP activity and proliferation potential, matrix mineralization did not decrease significantly with increases in donor age (slope = −0.0046 ± 0.0065, Figure 3c) when comparing all donors (irrespective of fat tissue location). Similar to AP activity, we observed only a slight increase in matrix mineralization (slope = 0.0215 ± 0.0160, insignificant, Figure 3d) with increases in donor BMI (regardless of fat tissue location). There was also no significant difference in matrix mineralization in Ad-MSCs of female and male donors (Figure 3e).

### 2.4. The Expression of Runx2 and SP7 is the Highest in Ad-MSCs of the Abdomen, Thigh, and Hip

**Figure 4:**
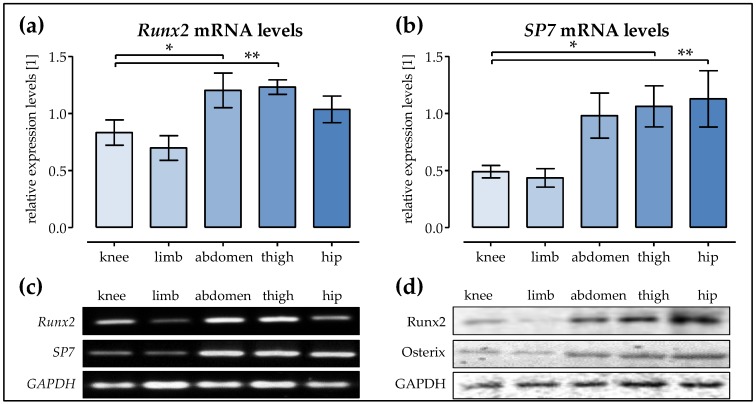
The expression of *Runx2* and *SP7* in Ad-MSCs varies depending on the donor site. Ad-MSCs derived from adipose tissue of the knee, limb, abdomen, thigh, and hip (*N* = 4 each) were osteogenically differentiated for 14 days. The relative expression levels of (**a**) *Runx2* and (**b**) *SP7* were determined by semi-quantitative RT-PCR. The PCR products were loaded twice (*n* = 2) to minimize the loading errors. The signal intensities were quantified with the help of the ImageJ software; (**c**) Representative images for the RT-PCR products from the pooled samples; (**d**) Representative images for the Western blot signals obtained from the pooled samples; * *p* < 0.05 and ** *p* < 0.01 as indicated.

The expression of the key osteogenic transcription factors *Runx2* (early) and *SP7* (late, encoding for osterix) was determined by semi-quantitative RT-PCR on day 14 of differentiation. Densitometric analysis (for a representative figure from the pooled samples see Figure 4c) revealed that, overall, the highest *Runx2* levels were observed in Ad-MSCs of the thigh, followed by Ad-MSCs of the abdomen. Interestingly, Ad-MSCs of the hip showed slightly lower *Runx2* expression levels. Ad-MSCs of the knee and the limb showed significantly lower *Runx2* expression (~0.6-fold, Figure 4a). Expression of *SP7* in Ad-MSCs of the abdomen was approximately twice as high as in Ad-MSCs derived from the knee and limb. The highest SP7 levels were observed in Ad-MSCs of the thigh and hip (Figure 4b). These changes were confirmed at the protein level by Western blot, using pooled samples for each donor site (Figure 4d).

### 2.5. The expression of the Stem ell Factors Oct4α, Lin28A, and Nanog is the Highest in Ad-MSCs of the Hip and Thigh

**Figure 5:**
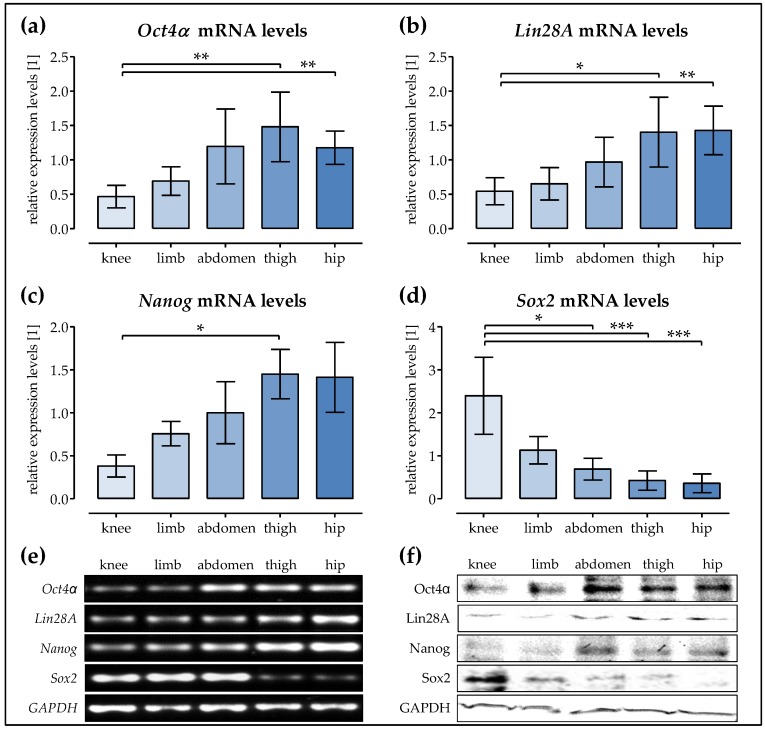
The expression of stem cell factors in Ad-MSCs varies depending on the donor site. The expression levels of the stem cell factors (**a**) *Oct4α*, (**b**) *Lin28A*, (**c**) *Nanog*, and (**d**) *Sox2* were determined by semi-quantitative RT-PCR in Ad-MSCs derived from adipose tissue of the knee, limb, abdomen, thigh, and hip (*N* = 4 each). The PCR products were loaded twice (*n* = 2) to minimize the loading errors. The signal intensities were quantified with the help of the ImageJ software. (**e**) Representative images for the RT-PCR products from the pooled samples; (**f**) Representative images for the Western blot signals obtained from the pooled samples; * *p* < 0.05, ** *p* < 0.01, and *** *p* < 0.001 as indicated.

In order to possibly explain the observed changes in the osteogenic differentiation potential, we determined the expression of stem cell factors in the Ad-MSCs. The expression levels of *Oct4α*, *Lin28A*, *Nanog*, and *Sox2* were determined by semi-quantitative RT-PCR before osteogenic differentiation (day 0). Densitometric analysis (for a representative figure from the pooled samples see Figure 5e) revealed the highest *Oct4α* levels in Ad-MSCs of the thigh, followed by Ad-MSCs of the hip and abdomen. Ad-MSCs of the knee and limb showed significantly lower *Oct4α* expression (Figure 5a). A similar pattern was observed for *Lin28A* expression. The highest expression levels of *Lin28A* were observed in Ad-MSCs of the thigh and the hip. Ad-MSCs from the abdomen showed slightly lower expression levels. The lowest expression levels for *Lin28A* were observed in Ad-MSCs from the limb and knee (Figure 5b). For *Nanog* expression levels, the effect was even more pronounced. The highest expression levels of *Nanog* were observed in Ad-MSCs of the thigh and the hip, followed by Ad-MSCs from the abdomen and limb. Ad-MSCs from the knee showed the lowest expression of *Nanog* (Figure 5b). Interestingly, the expression of *Sox2* was the highest in Ad-MSCs from the knee. Ad-MSCs from the limb showed moderate *Sox2* expression. *Sox2* expression levels were further reduced in Ad-MSCs from the abdomen and barely detectable in Ad-MSCs from the thigh and hip (Figure 5d). These changes were confirmed at the protein level by Western blot, using pooled samples for each donor site (Figure 5f).

## 3. Discussion

The current standard of care for filling large bone defects is the transplantation of autologous or allogenic bone graft tissue. This provides the bone defect site with a sufficient number of potent MSCs to stimulate bone regeneration. Therefore, for tissue engineering, B-MSCs derived from bone marrow aspirates are commonly used. Animal models could show that undifferentiated B-MSCs have a stronger potential for bone formation than Ad-MSCs [14]. However, as harvesting bone marrow is strictly limited in quantity and represents a painful and sometimes risky procedure for the patient, adipose tissue as a possible resource for autologous MSCs should not be excluded [10,13]. Our data show that Ad-MSCs can be obtained from various subcutaneous adipose tissues. Its harvesting (even in larger amounts) bears only little risk for the patient compared to bone marrow grafting, as it can be obtained by minimally invasive surgical techniques [11].

However, the bone tissue newly formed by tissue engineering often lacks mechanical stability, especially when using undifferentiated MSCs as the biologically active component [17]. Prior in vitro cell conditioning, however, requires special approval. Thus, the used scaffolds need to support the undifferentiated cells to either allow direct osteogenesis or secrete paracrine factors. A modification of the in vitro differentiation protocol was able to show that the replacement of dexamethasone by cholecalciferol significantly improved the osteogenic differentiation of Ad-MSCs [12]. The supplementation of the culture medium with platelet lysate or platelet-rich plasma also improved the osteogenic differentiation of Ad-MSCs [14,22,23]. Also, the cultivation on three-dimensional scaffolds further improved the osteogenic properties of Ad-MSCs [24,25], amongst others, through the control of cell agglomeration [26]. Furthermore, co-cultivation with endothelial cells, vascular cells, or osteoblastic cells improved the osteogenic differentiation of Ad-MSCs [27,28,29]. Furthermore, the osteogenic differentiation of Ad-MSCs is regulated by oxygen saturation in the medium [30]. This shows the strong responsiveness of Ad-MSCs to the local environment. Thus, tissue engineering may take advantage of these factors.

However, the literature also shows that there are factors that cannot be influenced by autologous cell therapy. For example, a time-consuming in vitro cell expansion may reduce the osteogenic differentiation potential of the cells, which might be due to epigenetic changes in the cells [18,19]. Similar mechanisms were observed with respect to an increasing donor age [20,21]. In addition, the initial weight or BMI of the donor may have a decisive influence on the osteogenic differentiation of isolated MSCs, particularly in Ad-MSCs derived from subcutaneous adipose tissue [31]. Our data clearly show that the donor site of the subcutaneous adipose tissue used for cell isolation has a significant influence on the proliferation and osteogenic differentiation potential of the isolated Ad-MSCs. Ad-MSCs derived from limb, abdomen, and knee continue to proliferate despite the presence of differentiation stimuli. However, the development of osteogenic properties, such as AP activity and matrix mineralization, was more pronounced in less proliferative Ad-MSCs derived from hip and thigh.

In the Ad-MSCs with the strongest osteogenic characteristics, the expression of the key osteogenic transcription factors *Runx2* and *SP7* was increased compared to more proliferative Ad-MSCs. Interestingly, *Runx2* levels were the highest in Ad-MSCs of the thigh and abdomen, and not the hip. It was reported that the early osteogenic transcription factor *Runx2* was reduced in favor of a faster induction of the late osteogenic transcription factor *SP7* [32]. This is in line with our data, showing the highest *SP7* expression levels in Ad-MSCs from the hip.

Within each group, a tendency towards an age-dependent decrease in osteogenic characteristics was visible, which is in line with our earlier observations [21]. However, comparing all samples with each other (regardless of the donor site) the observed effects were not age- or gender-specific. Furthermore, the donor BMI did not show a significant influence on the cells’ proliferation and differentiation properties. This is supported by the fact that some of the donors provided adipose tissue from multiple donor sites, and the observed effects were pronounced in direct comparison, thus precluding donor variability.

In order to more closely investigate the differentiation capacity of the Ad-MSCs, the expression levels of the stem cell factors *Oct4α*, *Lin28A*, *Nanog*, and *Sox2* was determined. Ad-MSCs derived from the hip, thigh, and abdomen, which all displayed good osteogenic characteristics, showed the highest expression levels of *Oct4α* and *Nanog*, which are described as core transcription factors for the regulation and maintenance of pluripotency in embryonic stem cells [33,34]. Tsai and co-workers have shown that the osteogenic differentiation capacity of MSCs decreases with the increase of time in culture. This effect was shown to be regulated by the *Oct4α* and *Nanog* expression [35]. In our earlier report, we have shown a similar trend dependent on the donor age [21]. However, this report focused on Ad-MSCs derived exclusively from the abdomen. In our present study, the effect of the donor site was more pronounced than the effect of the donor age.

*Lin28A* has been reported to promote cell proliferation in embryonic stem cells and breast tumor cells [36,37]. In our earlier work, its expression also decreased with the increase of the donor age, which positively correlated with the decreased proliferative capacity in these cells [21]. Interestingly, in this study, its expression was also the highest in Ad-MSCs derived from the hip and thigh, and not in the more proliferative Ad-MSCs from the knee, limb, or abdomen. As these Ad-MSCs were obtained from donors within a comparable age range, the effect of the donor site predominated.

Additionally, the basal expression levels of the stem cell factor *Sox2* were not comparable to the those of the other stem cell factors investigated. The highest basal expression of *Sox2* was observed in Ad-MSCs from the knee. Sox2 has been reported to regulate the proliferation and multipotency of stem cells in dependence of the cell density [38]. The over-expression of *Sox2* in c3h10t1/2 mouse embryonic stem cells effectively blocked their osteogenic differentiation [39]. This supports our observation that Ad-MSCs from the knee showed the least osteogenic characteristics. Ad-MSCs from the limb and the abdomen, showing strong proliferation despite the presence of osteogenic stimuli, showed moderate Sox2 expression levels. The lowest *Sox2* expression levels were observed in Ad-MSCs from the thigh and the hip, which showed the best osteogenic characteristics, suggesting that decreased *Sox2* expression levels may be pivotal for osteogenic differentiation. This is supported by the work of Park et al. showing that the depletion of Sox2 promotes osteogenic differentiation in mesenchymal stem cells from various tissues [40]. While this work proposes that Sox2 might inhibit Wnt signaling by inducing the expression of its inhibitor dickkopf-1, the work of Marcellini et al. proposed a direct interaction of Sox2 with its transcription factor β-catenin [41]. Furthermore, the work of Seo et al. showed that the over-expression of Sox2 not only inhibited the osteogenic differentiation of B-MSCs, but also favored their adipogenic differentiation via PPARγ [42].

## 4. Materials and Methods

### 4.1. Ethics Statement and Patient Information

All experiments were carried out in accordance with the Declaration of Helsinki (1964). For this study, cells were derived from adipose tissue from patients treated in our level 1 trauma center. According to the corresponding ethical vote (385/2012BO2), tissue was only harvested after medical consultation and written patient consent. The performed orthopedic/trauma surgery did not undergo chances due to the harvesting procedure. This means that the patients had no additional risk for complications, which is in line with earlier reports [43]. Neither patients not able to give their consent nor patients with viral or bacterial infections were included in the study. Information on the donors is summarized in Table 1.

### 4.2. Isolation, Expansion, and Differentiation of Primary Human Ad-MSCs

**Figure 6:**
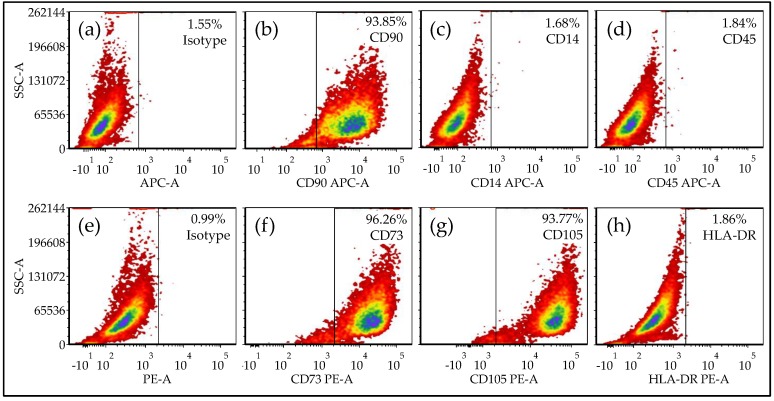
CD-marker expression in Ad-MSCs in passage 3. Ad-MSCs were stained for CD markers in passage 3. The analysis was done by flow cytometry as described [44] and is presented as scatter blot. Representative images for: (**a**) Isotype control for APC (allophycocyanin) secondary antibody. Ad-MSCs stained for (**b**) CD90-APC, (**c**) CD14-APC, and (**d**) CD45-APC. (**e**) Isotype control for PE (phycoerythrin) secondary antibody. Ad-MSCs stained for (**f**) CD73-PE, (**g**) CD105-PE, and (**h**) HLA-DR-PE.

Fat tissue (4–6 g per sample) was cut into mm-sized pieces using a scalpel and washed three to four times with phosphate buffered saline (PBS). For digestion, the pieces were incubated in 0.7% collagenase II solution (Biochrom, Berlin, Germany) for about 30 min at 37 °C. Collagenase digestion was stopped by adding culture medium (DMEM 4.5 g/L glucose, 10% FCS). After centrifugation at 600× *g* for 10 min, the Ad-MSC pellet was re-suspended in culture medium and expanded to passage 3 (37 °C, 5% CO_2_). Flow cytometry revealed that in passage 3, Ad-MSCs were negative for CD14, CD45, and HLA-DR and positive for CD73, CD90, and CD105 (Figure 6), which is in line with our earlier reports [44]. Thus, in passage 3, the cells (10^4^ cells/cm^2^) were osteogenically differentiated for 14 days. The osteogenic differentiation medium (DMEM 4.5 g/L glucose, 1% FCS, 200 μM L-ascorbic acid 2-phosphate, 10 mM β-glycerol phosphate, 25 mM HEPES, 1.5 mM CaCl_2_, 5 μM cholecalciferol) was replaced every 3–4 days [12].

### 4.3. Sulforhodamine B (SRB) Staining

To determine the number of cells by protein amount, the cells were fixed with ice-cold ethanol for 1 h. The fixed cells were then stained with a 0.4% SRB solution (in 1% acetic acid) for 20 min. After washing five times with 1% acetic acid, the remaining bound SRB was dissolved with 10 mM unbuffered TRIS solution (pH ~10.5) and quantified photometrically at the wavelength of 565 nm. The cell numbers were determined using a cell-specific standard curve, which was created during the course of the experiment [27].

### 4.4. Alkaline Phosphatase (AP) Activity

To determine AP activity, the cells were incubated at 37 °C with the substrate solution (1 mg/mL *p*-nitrophenyl phosphate, 50 mM glycine, 1 mM MgCl_2_, 100 mM TRIS, pH 10.5). The formed *p*-nitrophenol was measured photometrically at the wavelength of 405 nm. The signal was normalized to the relative cell number as determined by SRB staining [45].

### 4.5. Matrix Mineralization

To analyze the mineralized matrix, the cells were fixed with ice-cold ethanol for 1 h. The ethanol was then removed by washing three times with tap water. For von Kossa staining, the cells were covered with 3% silver nitrate solution for 30 min. After washing three times with tap water, incubation with a developing solution (5% Sodium carbonate, 10% formaldehyde) and 5% sodium thiosulfate solution was performed for two minutes each. For Alizarin Red staining, the cells were covered for 30 min with a 0.5% Alizarin Red solution (pH = 4.0). Unbound Alizarin Red was removed by washing with tap water. The dried plates were scanned. For quantification, the bound Alizarin Red was dissolved with a 10% cetylpyridinium chloride solution and quantified photometrically at a wavelength of 562 nm [45].

### 4.6. Semi-Quantitative RT-PCR

Total RNA was isolated using the Trifast reagent (Peqlab, Erlangen, Germany) as indicated by the manufacturer; cDNA sysnthesis was performed from 2 µg total RNA using the First Strand cDNA Synthesis Kit from Fermentas (ThermoScientific, Karlsruhe, Germany). Gene expression changes were investigated by semi-quantitative RT-PCR using the KAPA2G Fast Ready Mix from Peqlab. PCR conditions are summarized in Table 2. PCR products were separated by agarose gel electrophoresis and visualized by ethidium bromide (geldoc, INTAS, Göttingen, Germany). Individual samples were run twice (*n* = 2) to reduce the variations caused by small loading differences. The signal intensities were quantified using the ImageJ software (NIH, version 1.49m) [31].

### 4.7. Western Blot

The cells were lysed in freshly prepared ice-cold RIPA buffer. Amounts of 40 µg of total protein (pooled samples, *N* = 5 per donor site) were separated by SDS page and transferred to nitrocellulose membranes. The membranes were blocked with 5% BSA in TBS-T for 1 h followed by overnight incubation at +4 °C with primary antibodies for RUNX2 (sc-10758, Santa Cruz Biotechnology, Heidelberg, Germany), Osterix (MAB7547/R&D Systems), Oct4α, Lin28A, Nanog, and Sox2 (2840, 3695, 4903, and 3579, Cell Signaling Technology, Frankfurt am Main, Germany) diluted 1:1000 in TBS-T. The next day, the membranes were incubated with the corresponding peroxidase-labeled secondary antibodies (1:5000 in TBS-T, Santa Cruz Biotechnology) for 2 h. GAPDH (G9545, Sigma-Aldrich, Munich, Germany) was used as a loading control. For signal development, the membranes were incubated for 1 min with ECL substrate solution. The chemiluminescent signals, detected by a CCD camera (INTAS), were quantified using the ImageJ software.

### 4.8. Statistical Analysis

Results are represented either as bar diagrams (mean ± 95% confidence interval) or as scatter diagrams. Each group consists of 11 or 12 donors (*N* = 11 or 12). All measurements were performed as quadruplicates (*n* = 4). The comparison of multiple groups was done using Kruskal–Wallis H-test, followed by Dunn’s multiple comparison test. The comparison of two single groups was performed using the Mann–Whitney *U*-test (2-sided). Statistical analysis was performed with the help of the GraphPad Prism Software (Version 5, El Camino Real, CA, USA). A *p* < 0.05 was considered as statistically significant.

## 5. Conclusions

In conclusion, we were able to show that Ad-MSCs can be efficiently differentiated to an osteogenic cell linage. However, the donor site of the adipose tissue is crucial, as it has a much greater impact on the proliferation and osteogenic differentiation potential of the primary human Ad-MSCs than age, BMI, and sex of the donors. The ability to acquire osteogenic characteristics went along with an elevated expression of *Oct4α*, *Lin28A*, and *Nanog*, as well as a reduced expression of *Sox2*. The best osteogenic differentiation potential was shown by Ad-MSCs derived from hip adipose tissue, followed by thigh and abdominal adipose tissue. Ad-MSCs derived from the knee and the limb showed the strongest proliferation. However, a good proliferation potential together with a measurable increase in osteogenic characteristics were shown by Ad-MSCs derived from abdominal adipose tissue. Thus, depending on the requirements (cell amount and differentiation state), the donor site of adipose tissue could be adapted to obtain primary human Ad-MSCs.

## Figures and Tables

**Table 1:** Donor information.

	Knee	Limb	Abdomen	Thigh	Hip
Number of donors	12	11	11	12	12
Sex (male/female)	4/8	2/9	5/6	3/9	5/7
age (years)	62.2 ± 5.6	60.6 ± 5.7	53.3 ± 3.0	63.4 ± 6.3	60.3 ± 6.1

**Table 2:** PCR conditions.

Gene	GeneBank ID [NM]	Forward Primer	Reverse Primer	Ta [°C]	Amplicon [bp]
*Runx2*	001015051	TGCCTAGGCGCATTTCAGGTGC	GGTGGTGGTGCATGGCGGAA	58	359
*SP7*	152860	CCCAGGCAACACTCCTACTC	GGCTGGATTAAGGGGAGCAAA	58	175
*Oct4a*	002701.4	AGTGAGAGGCAACCTGGAGA	GCCTCAAAATCCTCTCGTTG	59	180
*Lin28A*	024674.4	CCGAACCCCATGCGCACGTT	TTTGCAGGTGGCTGCGCCAAG	59	137
*Nanog*	024865.2	AACTGGCCGAAGAATAGCAA	ACTGGATGTTCTGGGTCTGG	59	175
*Sox2*	003106.3	CATGCACCGCTACGACG	CGGACTTGACCACCGAAC	62	152
*GAPDH*	002046.4	GTCAGTGGTGGACCTGACCT	AGGGGTCTACATGGCAACTG	56	420

Ta—annealing temperature.

## Abbreviations

Ad-MSCs	mesenchymal stem/stromal cells from adipose tissue
AP	alkaline phosphatase
BMI	body mass index
B-MSCs	mesenchymal stem/stromal cells from bone marrow
CD	cluster of differentiation
MSCs	mesenchymal stem/stromal cells
PBS	phosphate buffered saline
SRB	Sulforhodamine B
TBS-T	TRIS buffered saline containing tween-20
